# The phase of ongoing EEG oscillations predicts the amplitude of peri-saccadic mislocalization

**DOI:** 10.1038/srep29335

**Published:** 2016-07-11

**Authors:** Douglas McLelland, Louisa Lavergne, Rufin VanRullen

**Affiliations:** 1 Centre de Recherche Cerveau et Cognition, Université Paul Sabatier, Université de Toulouse, Toulouse, France; 2 CNRS, CerCo, Toulouse, France; 3 Laboratoire Vision, Action, Cognition, Université Paris Descartes, Sorbonne Paris Cité, Paris, France; 4 Laboratoire Cognitions Humaine et Artificielle, Université Paris 8, Paris, France

## Abstract

Our constant eye movements mean that updating processes, such as saccadic remapping, are essential for the maintenance of a stable spatial representation of the world around us. It has been proposed that, rather than continually update a full spatiotopic map, only the location of a few key objects is updated, suggesting that the process is linked to attention. At the same time, mounting evidence links attention to oscillatory neuronal processes. We therefore hypothesized that updating processes should themselves show oscillatory characteristics, inherited from underlying attentional processes. To test this, we carried out a combined psychophysics and EEG experiment in human participants, using a saccadic mislocalization task as a behaviourally measureable proxy for spatial updating, and simultaneously recording 64-channel EEG. We then used a time-frequency analysis to test for a correlation between oscillation phase and perceptual outcome. We found a significant phase-dependence of mislocalization in a time-frequency region from around 400 ms prior to saccade initiation and peaking at around 7 Hz, principally apparent over occipital electrodes. Thus the degree of perceived mislocalization is correlated with the phase of a theta-frequency oscillation prior to saccade onset. We conclude that spatial updating processes are indeed linked to rhythmic processes in the brain.

Regular saccadic eye movements are a fundamental characteristic of the human visual system. While these enable us to rapidly and efficiently sample visual scenes using the high acuity foveal region of the retina, they also present a major challenge to the system in building or maintaining a stable representation of the spatial world around us. Nonetheless, our perceptual experience is of exactly that, a stable spatial world. There has been extensive investigation of the mechanisms of updating responsible for this visual stability.

Notably, substantial evidence indicates a link with attention^1^,^2^. For example, Irwin used arrays of letters to test the transfer of information about object identity and location across saccades, finding that only 3 to 4 items were accurately transferred, and that items close to the saccade target had an advantage^3^. This advantage was attributed to the known covert shift of attention to the saccade target prior to execution of the saccade^4^. It has since been directly demonstrated that this attentional advantage can extend to cued items beyond just the saccade target region^5^,^6^.

At the neuronal level, this transfer of information across saccades has been related to predictive remapping, that is, the transient shifting of receptive fields such that for a brief period even prior to a saccade, a cell may respond to a stimulus in its future (post-saccadic) receptive field (reported in parietal^7^, frontal^8^ and extrastriate visual cortex^9^,^10^, as well as superior colliculus^11^). Again, a link to attention is apparent: for example, in the lateral intraparietal area (a key region, with around half of the neurons showing predictive remapping^7^), cells do not respond to all stimuli, but only to the most salient (newly appearing) or behaviorally relevant^12^. Returning to the behavioural level, it has been shown that attention itself (measured as an improvement in discrimination) is predictively remapped prior to saccades^13^. Note also that receptive field remapping is thought to be responsible for the perceptual distortions of space around the time of saccades^14^,^15^ (see our experimental paradigm below).

In parallel with the development of these ideas, in the field of neuronal oscillations substantial evidence has now accumulated to show that attention samples the world periodically^16^,^17^,^18^,^19^,^20^, at a frequency of 7–8 Hz. For example, the phase of an ongoing oscillation in the EEG signal (~7.1 Hz) just prior to presentation of a weak visual target was found to be predictive of stimulus detection^21^. When two stimuli were presented, with one cued for attention, the effect was found only for the attended side^17^. Taken together with the suggested role of attention in visual stability, this leads naturally to the hypothesis that the updating process should likewise show evidence of periodicity. Of course, even if the link between updating and attention is ultimately rejected, the question of the temporal dynamics of updating remains an interesting one.

Several recent studies, like those outlined above, have demonstrated a link between the phase of ongoing neuronal oscillations and behavioral outcomes (perception^17^,^21^,^22^ or reaction time^23^), and we apply the same logic in this case. That is to say, we reason that if a neuronal oscillation (of a specific frequency) is functionally important for a given process, it should confer some periodicity on that process, and there should be a correlation between the *phase* of that oscillation (just before the onset of the process) and the behavioral outcome.

In this study then, we tested for correlations between oscillation phase, measured with 64-channel EEG recordings, and the outcome of the spatial updating process. For the latter, we used the phenomenon of “peri-saccadic mislocalization” as a proxy measure: perceptual mislocalization of a briefly flashed probe just prior to the execution of a saccade is supposed to reflect faulty remapping due to the absence of reliable pre- and/or post-stimulus representations of the probe^14^,^15^,^24^. For successive trial repetitions, a different amount of perceived mislocalization therefore indicates quantitatively different outcomes of the remapping process. By evaluating whether these different outcomes are associated with different pre-stimulus phase distributions (for any oscillatory signal), we were able to test whether spatial updating is periodic, and at what frequency. We found that the phase of a theta-frequency oscillation (~7 Hz) prior to saccade onset was correlated with the perceptual outcome, supporting the idea that updating is a periodic process.

## Results

### Peri-saccadic mislocalization

Our hypothesis concerns the role of spontaneous, ongoing oscillatory activity (as opposed to activity driven by or locked to external events). Therefore, a key requirement in our EEG-based paradigm is that the timing of the probe to be (mis) localized should be fully unpredictable. In the classic peri-saccadic mislocalization paradigm, a cue signals that the saccade should be launched, and the probe appears within a fixed time window after the cue^15^,^24^. In this case, the transient activity associated with the cue could reset oscillatory brain activity, making it impossible to isolate spontaneous oscillations in the critical time period. On the other hand, it is not practically feasible to use randomly timed probe flashes alongside spontaneously generated saccades because too small a fraction of probes will occur in the key time range for mislocalization (less than ~100 ms before saccade onset) by chance. Likewise, one cannot use a static probe instead of a briefly flashed one, because it will not be perceptually mislocalized^25^. Our solution, inspired by existing work^25^,^26^, was to use a moving probe in the form of a falling dot, with participants instructed to launch a saccade when the probe entered a demarcated area just above the level of the fixation and saccade target points. Thus, there was no sharply timed event that might reset oscillatory activity, and despite the ongoing nature of the stimulus, mislocalization was perceived.

**Figure 1:**
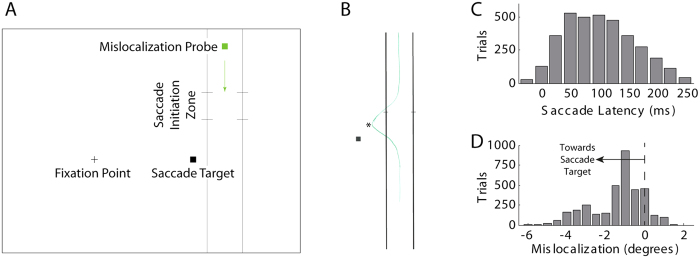
(****A****) Stimulus paradigm. The screen background was not white as shown here, but rather a dark red. Trials began when participants fixated the point at the left of the screen. The mislocalization probe appeared (all other elements were already onscreen), and fell towards the bottom of the screen. Participants were instructed to make a saccade to the saccade target point from around the time when the mislocalization probe passed through the demarcated zone. The mislocalization probe subsequently disappeared, and participants had to report the maximum horizontal extent (in either direction, but typically towards the saccade target) of any perceived deviation of the probe (which we take as a measure of mislocalization) at the time of the saccade. (**B**) Example sketch of one subject’s typical perceptual experience of the probe trajectory. The measured variable reported by subjects on each trial was the peak horizontal displacement (indicated by a star on the plot). (**C**) Histogram of saccade latency (all subjects, n = 12), relative to the time at which the mislocalization probe entered the saccade initiation zone. (**D**) Histogram of reported mislocalization (all subjects, n = 12), negative numbers indicating a perceived leftwards deviation (i.e. towards the saccade target, against the direction of the saccade).

The experimental paradigm is illustrated in Fig. 1A. Trials were initiated when subjects fixated a point at the left of the screen (detected by eye tracking). At that time, the probe appeared on the right, falling vertically at a rate of 10.2°/sec. To give subjects sufficient preparation time, the probe actually started in the lower part of the screen, fell off the bottom and reappeared at the top, still falling. Subjects were instructed to launch a saccade to the target point at around the time the probe passed through the demarcated area on the screen, but without this timing being strictly locked to a particular event; i.e. to the extent possible, saccades were spontaneously generated by subjects. Some subjects (n = 12; further details in Methods) reported seeing a sideways deviation of the probe at the time of the saccade (one subject’s impression in Fig. 1B), and their task was to report the peak horizontal extent of this deviation, using a mouse click at the end of the trial.

Consistent with the requirement for spontaneously generated saccades, the latency from the time of the probe entering the ‘saccade region’ to saccade launch was variable, both within and across subjects (Fig. 1C; mean latencies within subjects ranged from 37 to 187 ms, standard deviations, S.D., from 25 to 47 ms; mean S.D across subjects of 37 ms). Likewise, subjects perceived varying amounts of probe mislocalization (Fig. 1D), with mean deviations from −3.42° (leftwards) to +0.36° (rightwards) and standard deviations from 0.17° to 1.04° (mean S.D. across subjects 0.50°). Mislocalization was not dependent on saccade latency (significant linear correlation in only 3/12 subjects, with 2 positive and 1 negative correlation, and r-squared always less than 0.08). This provides reassurance that any correlations between EEG activity and mislocalization do not arise spuriously as a result of both being time-locked to some other event (e.g. the probe appearing initially, or entering the top of the screen or the saccade region).

### Correlation with EEG phase

We used a time frequency analysis to test for any correlations between behavioural outcome and the phase of oscillatory activity close to the time of the saccade, quantified as phase-opposition (see Methods for details).

**Figure 2:**
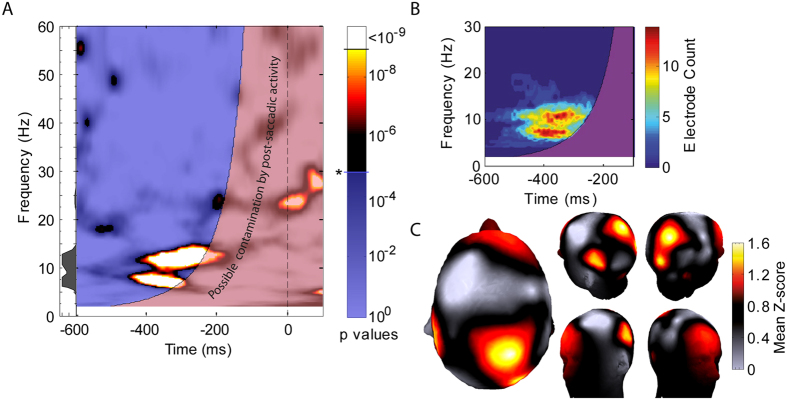
(****A****) Time-frequency plot of pre-saccadic phase-opposition between trials with different perceptual outcome, averaged across all participants and electrodes. Significance (p) values are plotted (fixed effects analysis, see methods). Empirical values were compared directly to a large set of surrogate distributions (10^9^ repeats), setting a limit on the minimum p-value that can be measured. For equivalent parametrically estimated values, see Supplementary Figure S1. The transparent red region around time-zero indicates the window of influence of the wavelet used for the time-frequency analysis, and thus susceptible to contamination from the saccade event at time zero. For the remainder of the plot, a transparent blue layer has been set at the 0.05 significance level (Bonferroni-corrected for the number of time and frequency comparisons), to emphasize higher values. The small area plot at the left hand side shows the profile of significance values across frequencies, measured at the time of the highest value (−356 ms). (**B**) Time-frequency plot, showing the outcome of a random-effects style analysis to test for regions of significant phase locking, corrected for multiple comparisons using a cluster-based technique (see Methods). The plot shows a count of the number of electrodes (out of 64 total) having a significant cluster incorporating the relevant time-frequency point. We focus on a smaller frequency range for greater detail. No significant clusters were apparent outside of this range. (**C**) Topographic plots showing the distribution of phase opposition hotspots for the region of interest identified above (for each electrode, phase-opposition z-scores averaged across all time-frequency pixels that showed significance over more than two electrodes).

Figure 2A presents a time-frequency plot of phase-opposition between trials with different perceptual outcome (low, medium or high mislocalization) averaged over all participants and electrodes, showing the significance of the measured phase-opposition relative to a surrogate distribution (fixed-effects analysis, see Methods). Because of the saccade event at time zero and the finite time window of the wavelet used for time-frequency analysis (longer at lower frequencies), a region around zero-time should be considered susceptible to contamination by muscular eye-movement artefacts–the transparent red region in the plot. For the remainder of the plot, a transparent blue layer has been set at the significance level (p = 0.05, Bonferroni-corrected for the number of time and frequency points tested) to emphasize any peaks in p-value higher than this. This level of correction is overly conservative, but nonetheless a region (or perhaps, pair of peaks) of significant phase opposition is apparent in the theta/alpha frequency bands, from around 6 to 13 Hz, and in the period 200 to 400 ms before saccade onset. Note that the significance of these data was estimated by direct comparison to a very large set of surrogate values (10^9^) avoiding the need to assume a normal distribution, but setting a lower limit on the p-values that can be estimated (p < 10^−9^). Because it provides a more detailed view of the peaks in significant phase opposition, an equivalent plot of parametrically estimated p-values is presented in Supplementary Figure S1.

Figure 2B shows a time-frequency plot of the same data tested at the electrode level (treating ‘subject’ as a random variable, see Methods), and a cluster-based technique^27^ to correct for multiple comparisons (across times, frequencies and electrodes). Again, the analysis reveals a time-frequency region of significant phase opposition: 6 to 13 Hz, 200 to 400 ms prior to saccade initiation (in a few electrodes, the significant cluster extends slightly further back in time, or extends to include slightly higher frequencies; however, no discrete clusters entirely separate from this region were apparent). Taking the time-frequency region identified by this analysis, Fig. 2C shows the topographical distribution of phase opposition values (z-scores from the fixed effects analysis averaged across the identified time-frequency region, characterized as any point included in significant clusters on more than two electrodes), revealing that the major contribution arises from occipital, parietal and right frontal electrodes, consistent with the known involvement of these regions in attention, eye movements and remapping. The probe was always presented in the right visual hemifield and so, to the extent that the data show lateralization, the involvement of left parietal areas seems predictable, but the involvement of right frontal and parietal areas is actually also consistent with the recognized dominance of this hemisphere in spatial attention^28^.

We also generated similar topographical data to Fig. 2C, but treating the two apparent peaks in phase opposition separately (i.e. averaging across significant time-frequency points separately for frequencies above or below 9 Hz). However, the two distributions generated looked similar, confirmed by a significant correlation between them (Spearman’s correlation of z-scores across electrodes; rho = 0.3092; p = 0.0132). We therefore chose to treat the results as showing a single time-frequency region of interest (possibly a fundamental frequency and first harmonic)–at least, on the basis of the current results we see no argument for two distinct oscillations, although clearly we cannot rule out this possibility.

**Figure 3:**
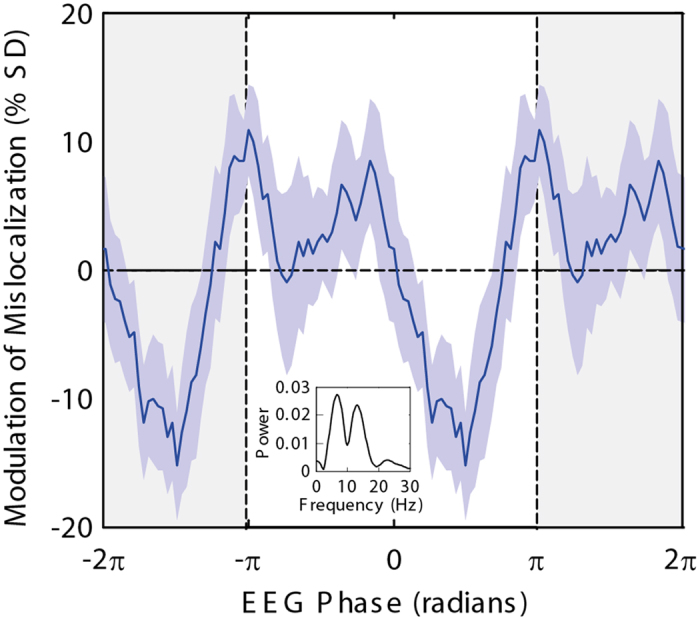
Modulation of mislocalization as a function of EEG phase, calculated for the individual time-frequency point showing highest phase opposition (electrode POz, −358 ms, 6.6 Hz). The plot shows the average (±S.E.M) of smoothed curves fit to the data for each subject, extended to two oscillation cycles for clarity. The inset shows the power spectrum of this average curve, with a peak at the fundamental frequency of 6.6 Hz as expected, and a second peak at the first harmonic frequency (13.2 Hz) emphasizing the non-sinusoidal nature of the curve.

To illustrate the results of this time-frequency analysis in a more intuitively accessible way, Fig. 3 shows the extent to which the amount of perceived mislocalization is correlated with oscillation phase, calculated for the single time-frequency point of maximum influence (electrode POz, −358 ms, 6.6 Hz). For each subject, we normalized mislocalization data by converting to z-scores, fitted a smoothed curve (Matlab ‘smooth’ function using a 2^nd^-degree Savitsky-Golay filter, equivalent to a weighted moving average but allowing for the irregular x-spacing of the data points; span set to include 30% of data points, or approximately a 45 ms window) to the mislocalization vs phase data, then averaged these curves across subjects. Note that this figure is intended for easier understanding only, not as any further evidence of the correlation between mislocalization and oscillation phase, because we selected these data from the most significant point in Fig. 2. That said, the figure does helpfully allow some estimate of the extent to which perceived mislocalization is modulated according to oscillation phase, specifically, by about ±10% of the mislocalization standard deviation. This value likely reflects a lower estimate of the true modulation, in that no realignment of ‘best’ or ‘worst’ phases across subjects was included here. The inset panel in Fig. 3 presents the power spectrum of the averaged curve, showing a strong peak at the fundamental frequency (6.6 Hz) predetermined by the analysis, and a second peak at the first harmonic frequency (13.2 Hz), emphasizing the non-sinusoidal nature of the curve. This non-sinusoidal modulation of mislocalization with phase may account for the pair of peaks at comparable frequencies in the time-frequency plots (Fig. 2A,B). For completeness, equivalent plots for electrodes F8 (right frontal) and P7 (left parietal), along with time-frequency plots of phase opposition (equivalent to Fig. 2A but for single electrodes) are shown in Supplementary Figure S2.

A second approach to visualize the data in an accessible way is to look at the EEG data in the original time domain, as an event-related potential (ERP) time locked (but prior to) saccade onset, averaged separately for different behavioral outcome groups. Two difficulties must be confronted in this case: a) the phase opposition analysis used above is, by design, indifferent to the amplitude of the various frequency components in the EEG signal, but the ERP is not and thus high amplitude activity at other frequencies can obscure the frequencies of interest; b) the absolute phase of effects is not necessarily constant across subjects (because of subtle differences in the spatial arrangement of cortex and electrode placement across subjects; the simplest working assumption is that the underlying neuronal activity itself would show a consistent phase relation)–again, the phase opposition analysis is immune to this factor (because it works on within-subject phase differences, not absolute phase) whereas the ERP is not, and so averaging across subjects can actually conceal real effects in this case. Indeed, ERPs for the different behavioral outcomes, averaged across subjects, showed only minor differences, small in comparison to the amount of intersubject variance (data not shown). However, individual subject ERPs proved to be helpful, with clear oscillatory activity and a striking phase difference between the low and high mislocalization trial groups. Data from 6 subjects are shown in Supplementary Figure S3. Note that, although the ERPs are visually dominated by high amplitude oscillatory activity at around 10 Hz, Fourier analysis reveals that the 7 Hz phase opposition effect revealed in our main analysis is also present.

## Discussion

We have demonstrated that the phase of ongoing oscillatory brain activity in the theta/alpha frequency bands is correlated with the outcome of the spatial updating process around the time of saccades.

We take this correlation to demonstrate a functional role of neuronal oscillations in the spatial updating process, in which case the strongest correlation should naturally be found for time points at or just before the time of the saccade, but extending back in time as a function of the frequency-stability of the oscillations. With this in mind, we interpret the timing of the correlation we find (at 400-300 ms pre-saccade) not as an indication of the full dynamics of these processes, but as a correlation at the latest time point available to our analysis (given the finite time window of wavelet analysis and the contamination of the EEG data starting from the time of the saccade). This would suggest that a slightly stronger correlation might be expected closer to saccade onset if that time period were accessible to our analysis.

To further interpret these results, it is necessary to consider the underlying mechanisms. On the spatial updating side, it has been suggested that peri-saccadic mislocalization indexes errors in saccadic remapping^15^,^24^, widely accepted as the key physiological-level mechanism underlying spatial updating^29^. Thus our findings suggest that either:

(a) Saccadic remapping is itself a rhythmically modulated process

and/or

(b) Saccadic remapping inherits the rhythmic characteristics of some closely associated process, with attention as a likely candidate^1^

Consistent with the latter proposal, the 7 Hz oscillation that we identify here matches the frequency of that previously described in attentional experiments^17^. It is further worth noting that there may be no real distinction between the two conclusions above: it has been suggested that what is remapped is not actually the content of the visual scene but rather spatial pointers to attended objects^30^, and that these ‘attention pointers’ are in fact key players in the attentional process itself.

## Methods

### Participants

32 participants (11 male) volunteered after giving written informed consent. Perceived saccadic mislocalization is less robust in the current paradigm than in the classic paradigm^24^ with some individual variability, and so participants underwent a preliminary psychophysical test to determine whether they perceived mislocalization. 12 participants (4 male) experienced measureable mislocalization and were retained for further testing. All participants had normal or corrected to normal vision. All experiments were carried out in accordance with the protocol approved by the Centre National de la Recherche Scientifique ethical committee.

### Stimuli and procedure

Stimuli were presented on a 150 Hz cathode ray tube monitor. The experiment was run using Matlab (Mathworks) and the Psychophysics Toolbox extensions^31^,^32^.

Stimuli were presented against a dark red background (CIE xyY coordinates, 0.524, 0.354, 5.78). During each trial, several objects (black, CIE xyY: 0.507, 0.257, 0.02) were continuously on-screen (Fig. 1A): a) a fixational start point on the left hand side (random position from 9.9° to 6.4° left of screen centre) b) a saccade target towards the right (always 10.6° from fixation point) c) a pair of vertical tramlines (1.5° and 5.3° to the right of the saccade target) to aid in perception and reporting of the mislocalization probe and d) a pair of small horizontal markers on each of these tramlines demarcating a rectangular zone from 4.3° to 7.2° above the level of the start and saccade target points. Trials were launched by participants fixating the start point, detected using an eyetracker (see below). Then the mislocalization probe, a small pale green (CIE xyY: 0.310, 0.522, 7.31) dot appeared 3.4° to the right of the saccade target (mid way between the two vertical tramlines). The probe position relative to the saccade target was chosen in order to produce leftwards mislocalization, against saccade direction, as expected from saccadic compression^24^ or remapping^26^. The probe moved down the screen (speed 10.2°/sec), starting in the lower portion, disappearing from the bottom and reappearing at the top. Perceived mislocalization was found to be enhanced when the probe had a rather vague spatial position, achieved by introducing a random element to the vertical position of the probe: rather than descending smoothly, on each frame the “mean” position of the dot descended by a fixed amount but the actual vertical position varied around this as a normal distribution with standard deviation 0.76°. Given the high frame rate, the resulting appearance was of a blur or flickering cloud of dots falling down the screen, although actually only a single dot at a precise location was present at any given time. Participants were instructed to perform a saccade to the saccade target at approximately the time that the probe traversed the demarcated rectangle (thus the probe was approximately level horizontally with the saccade target at the time of the saccade). The probe continued into the lower portion of the screen, then disappeared. Participants reported the maximum spatial extent of any perceived horizontal deviation of the probe at the time of the saccade using a single mouse click at the perceived location on the screen. Participants were then free to pause and relax before initiating the next trial by fixating the start point. Six blocks of 60 trials were carried out by each participant. At the end of the experiment, subjects were asked to sketch their perceptual impression of the probe trajectory on a diagram (Fig. 1B).

### Data Acquisition and Analysis

Eye position was continuously monitored using an IView system (SensoMotoric Instruments), to detect start point fixation and saccade timing. The system was calibrated at the start of each 60-trial block, using a 13 point calibration defined by an IviewX Matlab command. Calibration was checked at the beginning of each trial with an invisible boundary check in a 2°-side square centred on the fixation cross. Signals were digitized at 1 kHz.

Simultaneously, continuous EEG was acquired with a 64-channel ActiveTwo system (Biosemi). Electrodes were placed according to the international 10–20 system, mounted in an elastic cap. Vertical and horizontal electrooculograms were recorded using additional electrodes below the left eye and at the outer canthi of both eyes–these signals were used as an additional check to ensure good synchronization of the EEG and eyetracker signals. EEG signals were digitized at 1024 Hz, 24-bit A/D conversion.

Data were analyzed using Matlab (Mathworks) and the EEGLAB toolbox^33^, with eyetracker data imported and saccadic events detected automatically using the EYE-EEG plug-in for EEGLAB^34^,^35^ (detects saccades using the velocity-based approach described previously^35^). Data were converted to an average reference, downsampled offline to 256 Hz, and epoched from 1750 ms before to 1250 ms after the saccade time. Data were screened manually for unwanted eye movement and other artefacts. We rejected any trials in which there was not an initially stable eye position followed by a single clear saccade to the target. Note that although epochs extended past the time of the saccade, in further analysis, only pre-saccadic time points were of interest: the time course for classical flashed-stimulus perisaccadic mislocalization is from around 50 ms pre-saccade up to and including the saccade itself but not after^24^ and further, data after the onset of the saccade are too contaminated by muscular eye-movement artefacts to be useful.

Initial time-frequency analysis was carried out using the EEGLAB toolbox (timefreq function). Parameters were set to yield an analysis over a range of 60 discrete frequencies increasing logarithmically from 2 to 100 Hz, with filter length (Morlet wavelets) varying smoothly from 2 cycles at the lowest frequency to 20 cycles at the highest. This yields, for each trial, *k*, a complex vector representing the amplitude, *A*, and phase, φ of oscillatory activity at each time point, *t*, and frequency, *f*.


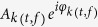


Phase locking (or intertrial coherence, ITC) quantifies the extent to which the phase of a given oscillation is consistent across trials (at a given time point relative to some time-locking event, in this case saccade initiation), and was calculated as described previously^36^. Briefly, for a given time/frequency point, for each trial, complex vectors were normalized to unit length (i.e. retaining only phase information), then averaged across trials. The length of the resulting vector indicates the extent of phase locking over trials.

### Phase Opposition

To assess any correlation between oscillation phase and behavioural outcome, we computed the phase opposition^37^, that is the sum of phase locking values calculated separately for trial groups separated according to behavioural outcome. If behavioural outcome is correlated with phase, then the sum of phase locking values across these trial groups will be higher than for an equivalent surrogate measure, the sum of phase locking values across equally sized groups with randomly assigned trials. In previous studies using this style of analysis^21^,^22^, it was typically natural to split trials into two groups because of the nature of the task (e.g. hits vs misses^21^,^22^, but see^23^ for an analysis using multiple groups). In the current study, because the behavioural measure, perceived mislocalization, is a continuous value, trials can be divided into an arbitrary number of groups. We divided trials into three equally sized groups (high, medium and low mislocalization), reasoning that this could potentially yield increased sensitivity to phase effects (consider, e.g. if mislocalization were a sinusoidal function of phase: groups of trials with high mislocalization or with low mislocalization would show strong phase concentration, towards the peak and the trough of the sinusoid respectively; however, trials with intermediate mislocalization would show poor phase concentration and so their inclusion in the high or low groups could dilute any existing effects), while still maintaining reasonable trial numbers (about 100 trials per group per subject, after elimination of trials due to EEG artefacts or inappropriate eye movements).

In order to assess the significance of measured phase opposition values, we then generated sets of 1000 surrogate phase opposition values for each subject (that is, trials were randomly reassigned to ‘high’, ‘medium’ or ‘low’ groups, and phase opposition values for each electrode and each time-frequency point were recalculated, and this was repeated 1000 times; we will refer to these as “within-subject surrogates” to distinguish them from the second level of “surrogate averages”, described below). This is necessary because ITC values (and therefore also phase opposition values) are never expected to equal zero, even when samples are drawn from a uniform random distribution of phases. Rather, they are always positive, but are expected to approach zero in a predictable fashion as the number of samples increases. The advantage of comparing the empirical phase opposition values to surrogate distributions (rather than mathematically predicted values for the number of samples) is that we can dispense with the assumption that the underlying distribution of phases (across all trials, before grouping for phase opposition) is random and uniform. Even if the EEG signal has some non-random phase relation to the time-locking event (in our case, the saccade), that non-randomness will be reflected in the surrogate measures, and any extra phase-dependency of behavioural outcome will still be apparent in the phase opposition measure.

There are then a number of statistical approaches available to compare empirical and surrogate data, taking into account the averaging across subjects and multiple comparisons across time- and frequency-points and electrodes^27^. We tried several approaches, and obtained consistent results across each. Here we present two:

First, to provide a general overview, we measured the grand average phase opposition value across all subjects and electrodes (separately for each time-frequency point). Each empirical average was compared to a distribution of identically calculated “surrogate averages”. These latter were generated by averaging across randomly selected within-subject surrogates and repeating this process many times (i.e. with 1000 within-subject surrogate values for each of *n* subjects, there are 1000^n^ ways that these could be combined across subjects). To avoid any assumptions about the form (normal or otherwise) of the distributions of surrogate averages, for our main analysis we generated 10^9^ values (independently for each time-frequency point), to allow a direct comparison to the empirical values. Additionally, because the surrogate distributions are not rejected by tests for normality, and because it reveals greater detail of the peaks in phase opposition, we include equivalent results calculated parametrically, that is, relative to the mean and standard deviation of the surrogate distributions (Supplementary Figure S1; see legend for further details). (Note that averaging EEG data is complicated by the fact that measures on different electrodes are not independent of each other. Our analysis compensates for this by making sure that the same non-independence is present in the surrogate averages: for each iteration, for a given subject the same random trial groups were picked for all electrodes).

Second, we present an analysis at the electrode level, treating subjects as a random variable (following the within-subjects style analysis described by Maris and Oostenveld^27^), essential if the results are to be generalized beyond the measured population. We took the median within-subject surrogate phase-opposition value (for each time/frequency/electrode point) as the ‘background’ condition to be compared to the empirically measured value. We then generated a surrogate distribution of mean values across subjects by permuting these pairs of values (i.e. background and empirical values systematically switched for each subject, for all possible combinations; 2^n^ possibilities for n subjects), to which the empirical values were compared. We controlled for multiple comparisons using a cluster-based method^27^,^38^: from the distribution of surrogate means, we identified the upper 5^th^ percentile of values (independently for each time/frequency/electrode point). We then applied this threshold to the full data set (empirical and surrogate values) and measured the largest (by pixel-count rather than sum of values^38^) supra-threshold cluster of adjacent points on each time-frequency map (64 electrodes times 2^12^ permutations). Returning to the empirical data, we report those clusters larger than the upper 5^th^ percentile of cluster sizes.

### Data Archive

The data from this study are available in a publically accessible archive hosted by the Open Science Framework, at: osf.io/ntx8a.

## Additional Information

**How to cite this article**: McLelland, D. *et al*. The phase of ongoing EEG oscillations predicts the amplitude of peri-saccadic mislocalization. *Sci. Rep*. **6**, 29335; doi: 10.1038/srep29335 (2016).

## Supplementary Material

Supplementary Information
