# Supplementary material for: The phase of ongoing EEG oscillations predicts the amplitude of peri-saccadic mislocalization
Source: Sci Rep. 2016 Jul 11;6:29335. doi: 10.1038/srep29335 (PMC4941415; doi:10.1038/srep29335)
Supplement: Supplementary Information [file srep29335-s1.pdf]

The phase of ongoing EEG oscillations predicts the amplitude of peri-saccadic mislocalization

Douglas McLelland, Louisa Lavergne & Rufin VanRullen

Supplementary Information.

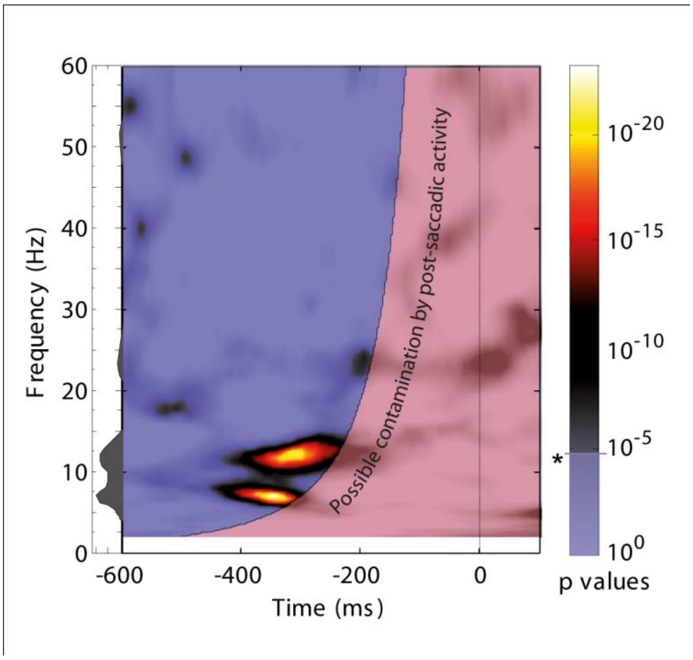

Figure S1. Time-frequency plot of pre-saccadic phase-opposition between trials with different perceptual outcome, averaged across all participants and electrodes, exactly corresponding to Figure 2a except that, rather than directly computing a very large surrogate population (as in Fig. 2a), here the significance of the empirical values was calculated relative to the mean and standard deviation of 1000 surrogate values, independently for each time-frequency point (valid under an assumption of normality for those distributions; Shapiro-Wilk tests repeated for each time-frequency point returned fewer than 10% $p < 0.05$, comparable to the rejection rate for randomly generated 'normal' data from the Matlab randn function, and on repeating the procedure with different sets of surrogate distributions, different time-frequency points were rejected each time, i.e. no time-frequency points were systematically rejected as non-normal). The transparent red region around time-zero indicates the window of influence of the wavelet used for the time-frequency analysis, and thus susceptible to contamination from the saccade event at time zero. For the remainder of the plot, a transparent blue layer has been set at the 0.05 significance level (Bonferroni-corrected for the number of time and frequency comparisons), to emphasize higher values. The small area plot at the left hand side shows the profile of significance values across frequencies, measured at the time of the highest value (-356 ms). A main peak is apparent at 7.0 Hz and a secondary one at 12.0 Hz.

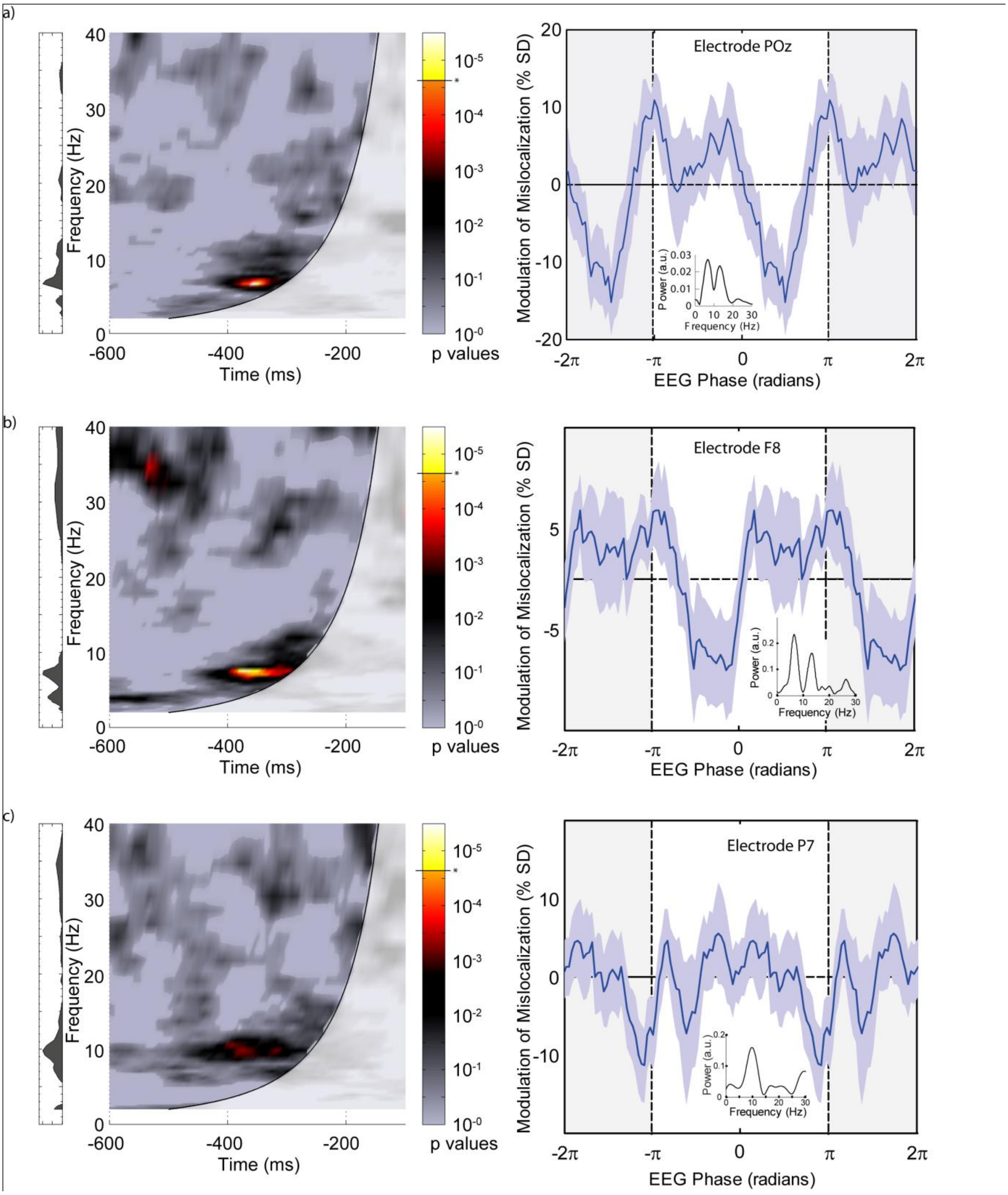

Figure S2. Phase opposition at individual electrodes. Phase opposition as a function of frequency, and time relative to saccade onset (left panels) and mislocalization as a function of phase (right panels; measured for the time and frequency of peak phase opposition), at individual electrodes, reflecting the parieto-occipital, frontal and parietal peaks of phase opposition apparent in the topographical plot (Figure 2c). a) Electrode POz (Central parieto-occipital region), peak at -358 ms, 6.6 Hz. b) Electrode F8 (right frontal region), peak at -373 ms, 7.5 Hz. c) Electrode P7 (left parietal region), peak at -373 ms, 9.8 Hz.

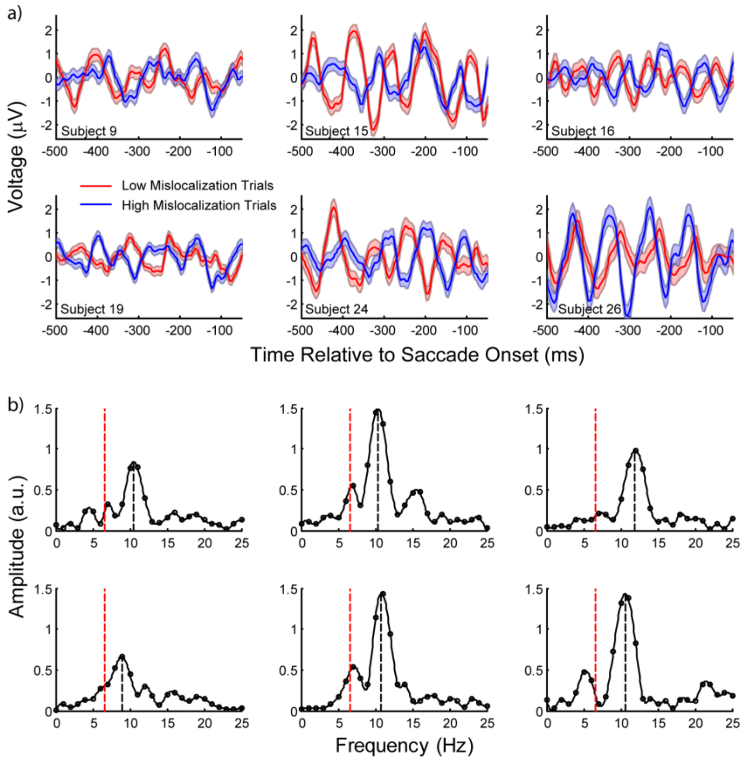

Figure S3. Examples of phase opposition in individual subject event-related potentials (ERPs). a). ERPs for a selection of individual subjects (electrode POz, bandpass filtered between 4 and 40 Hz; averaged across trials, traces show mean ± s.e.m. across trials), time locked to saccade onset (time zero), averaged separately for trials with low mislocalization and high mislocalization (for clarity, the medium mislocalization group is not shown). Note how the phase of oscillatory activity for the two groups is clearly misaligned. b) Power spectra of the difference between the trial group ERPs shown above (for the corresponding subjects). The black dashed line marks the frequency of peak power, the red dashed line is set for reference at 6.6 Hz, the frequency of the strongest phase opposition effect revealed in the main analysis. Note that, although this time domain analysis is dominated by alpha frequency effects (~10 Hz) our main analysis, which is immune to the amplitude of oscillatory activity, revealed an even stronger phase-opposition effect at slightly lower frequencies around 7 Hz (activity which is also apparent in the ERP-based spectra, albeit less apparent to the naked eye).
